# Comparison of Metabolites Variation and Antiobesity Effects of Fermented versus Nonfermented Mixtures of *Cudrania tricuspidata*, *Lonicera caerulea*, and Soybean According to Fermentation *In Vitro* and *In Vivo*

**DOI:** 10.1371/journal.pone.0149022

**Published:** 2016-02-05

**Authors:** Dong Ho Suh, Eun Sung Jung, Hye Min Park, Seung Hyung Kim, Sarah Lee, Yang Hee Jo, Mi Kyeong Lee, Gayoung Jung, Seon-Gil Do, Choong Hwan Lee

**Affiliations:** 1 Department of Bioscience and Biotechnology, Konkuk University, Seoul, Republic of Korea; 2 Institute of Traditional Medicine & Bioscience, Daejeon University, Daejon, Republic of Korea; 3 National Institute of Biological Resources, Incheon, Republic of Korea; 4 College of Pharmacy, Chungbuk National University, Cheongju, Republic of Korea; 5 Wellness R & D Center, Univera, Inc., Seoul, Republic of Korea; College of Tropical Agriculture and Human Resources, University of Hawaii, UNITED STATES

## Abstract

We used ultra-performance-liquid-chromatography with quadrupole-time-of-flight mass spectrometry to study the changes in metabolites in the mixture of *Cudrania tricuspidata*, *Lonicera caerulea*, and soybean (CLM) during fermentation. Additionally, the antiobesity effects of CLM and fermented-CLM (FCLM) were studied based on the analysis of plasma from high-fat diet (HFD)-fed mice. The levels of cyanidin and the glycosides of luteolin, quercetin, and cyanidin derived from *L*. *caerulea* were decreased, whereas the levels of luteolin and quercetin were increased during fermentation. Isoflavone glycosides and soyasaponins originating from the soybean were decreased, whereas their aglycones such as daidzein, glycitein, and genistein were increased. As for prenylated flavonoids from *C*. *tricuspidata*, these metabolites were decreased at the early stage of fermentation, and were increased at end of the fermentation. In terms of the functional food product, various metabolites derived from diverse natural products in CLM had complementary effects and demonstrated higher antioxidant and pancreatic lipase inhibition activities after fermentation; these activities were closely related to flavonoid aglycones including genistein, daidzein, glycitein, luteolin, and quercetin. In an *in vivo* experiment, several clinical parameters affected by HFD were improved by the administration of either CLM or FCLM, but there was a difference in the antiobesity effects. The levels of lysoPCs with C20:4, C16:0, and C22:6 were significantly attenuated by CLM administration, while the attenuated levels of lysoPCs with C20:4 and C18:2 were significantly restored by FCLM administration. These metabolites may explain the above-mentioned differences in antiobesity effects. Although only the changes in plasma lysophospholipids could not fully explain antiobesity effects between non-fermented and fermented plant mixtures from our results, we suggest that metabolomics approach could provide a way to reveal the metabolite alterations in the complex fermentation process and understand the differences or changes in bioactivity according to fermentation.

## Introduction

Recently, interest in the relation between diet and health increased the demand for functional food products, thereby increasing health care costs over the years. Beyond basic nutrition, these products provide health benefits and prevent the risk of certain diseases. Generally, a functional food product is a food with an additional function including health promotion or disease prevention by providing new ingredients or higher levels of known nutrients. To satisfy the high demand, numerous new products with various advantages have been introduced in the last few years [1,2]. In particular, fermented functional food products are gaining interest in food industries around the world. Fermentation of the raw materials has various advantages, including improvement of the health benefits by enhancing nutrients, by making the food more easily digestible than the original raw food, and by removing toxic chemicals via the fermentation process. In addition, various fermented natural products synergistically enhance the beneficial effects of diverse raw foods compared to the unfermented products [3–5].

Fermented soybean products, well-known fermented functional foods, are famed for their numerous health benefits including antioxidant activity, pancreatic lipase inhibition, and antiobesity effects *in vivo*, which are greater than those of the unfermented soybean [6–8]. Despite various studies on fermented soybean products, there is a lack of research on fermentation of soybean supplemented with other natural products in relation to the antiobesity effects. In nature, there are many little-known natural products with potential antiobesity effects. Among them, *Cudrania tricuspidata* has radical-scavenging, pancreatic-lipase-inhibitory, and anti-inflammatory activities and contains prenylated flavonoids and isoprenylated xanthones [9,10]. *Lonicera caerulea* has beneficial bioactivities: prevention of diabetes, cardiovascular diseases, and cancer, owing to various anthocyanins and polyphenolic compounds [11]. *C*. *tricuspidata* and *L*. *caerulea* possess effects related to antiobesity properties and contain new ingredients compared to the soybean alone. We hypothesized that fermentation of a mixture of *C*. *tricuspidata*, *L*. *caerulea*, and soybean (CLM) can produce a functional fermented food product because of enhanced antiobesity effects.

Mass spectrometry (MS)-based metabolite profiling is usually used to identify changes in metabolites due to the various activities of fermentation [12,13]. In addition, this approach has been frequently used to find biomarkers in obesity- and diabetes-related studies [14–17]. In the present study, we performed metabolite profiling to identify changes in metabolites in CLM during fermentation. In addition, we used ultra-high-performance liquid chromatography with quadrupole time-of-flight mass spectrometry (UPLC-Q-TOF-MS) to compare the differences in plasma metabolites between groups of mice receiving unfermented CLM or fermented CLM (FCLM), i.e., in the mouse model of high-fat diet (HFD)-induced obesity (we also compared *in vivo* antiobesity effects).

## Materials and Methods

### Chemicals and Reagents

Methanol, acetonitrile, and water were purchased from Fisher Scientific (Pittsburgh, PA, USA). Formic acid, potassium persulfate, 2,2′-azinobis(3-ethylbenzothiazoline-6-sulfonic acid) diammonium salt (ABTS), 6-hydroxy-2,5,7,8-tetramethylchromane-2-carboxylic acid (trolox), sodium carbonate, naringin, gallic acid, Folin & Ciocalteu’s phenol reagent, diethylene glycol, porcine pancreatic lipase, Tris-HCl buffer, *p*-nitrophenylbutyrate (*p*-NPB), dimethyl sulfoxide, and standard compounds were obtained from Sigma Chemical Co. (St. Louis, MO, USA).

### Preparation of the fermented-mixture samples preparation

**Fig 1:**
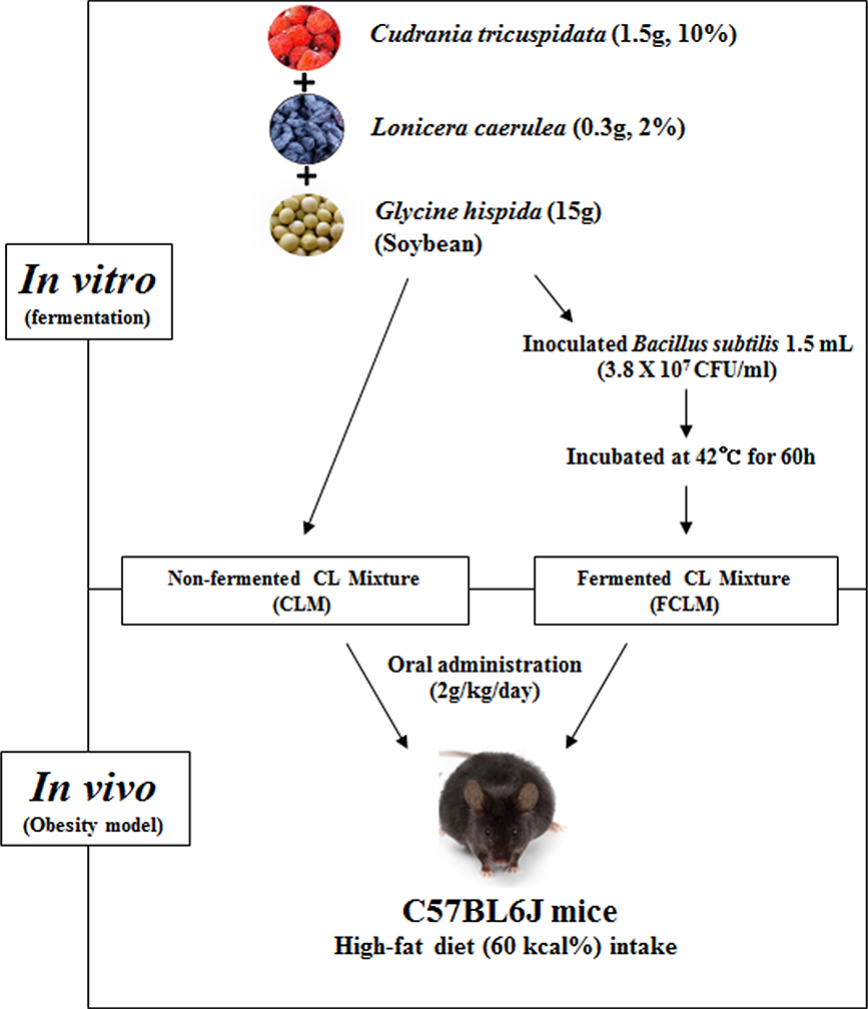
Schematic representation of the experimental procedures.

We collected and used *Glycine hispida* (soybean) and *L*. *caerulea* cultivated from a farm in Kraskino, Khasansky District, Primorsky Krai, Russia, as well as *C*. *tricuspidata* cultivated from a farm in Okchen, Chungcheongbuk-do, Republic of Korea. Whole soybean (250 g) was soaked overnight in 1 L of water at room temperature and then autoclaved at 121°C for 60 min. *L*. *caerulea* and *C*. *tricuspidata* were freeze-dried for 7 days and ground into a powder using a mortar and pestle. *Bacillus subtilis* were grown in nutrient broth for 1 day at 37°C in the dark with shaking (200 rpm). The sample preparation process is shown in Fig 1. Briefly, soaked soybeans (15 g) were crushed using a spatula in a Petri dish. The fine powders of *L*. *caerulea* (0.3 g) and *C*. *tricuspidata* (1.5 g) were mixed with the crushed soybeans. After that, *B*. *subtilis* was inoculated (1.5 mL) into the mixture, and the dish was incubated at 42°C. Samples were collected at regular intervals: 0, 8, 16, 24, 32, 40, and 60 h. After the fermentation, the samples were stored at temperatures below -80°C and then freeze-dried for 3 days.

Each dried FCLM sample (1 g) was extracted with 80% methanol (20 mL) by sonication for 15 min, and then shaken on a Twist Shaker (BioFree, Seoul, Korea) for 2 h at room temperature. After that, the extracts were centrifuged at 2,370 *g* for 10 min at 4°C (Hettich Zentrifugen Universal 320, Tuttlingen, Germany). The supernatant was passed through a 0.2-μm polytetrafluoroethylene (PTFE) filter and dried up in a speed vacuum concentrator (Biotron, Seoul, Korea). The final concentration of the analytes was 10 mg/mL in 80% methanol.

### Determination of total phenolic content (TPC) and total flavonoid content (TFC) during the fermentation

TPC was quantified as described by Singleton et al. [18] with some modifications. To measure TPC, 20 μL of each sample was combined with 100 μL of 0.2N Folin & Ciocalteu’s reagent in 96-well plates and incubated for 6 min at room temperature in the dark. Then, 80 μL of a 7.5% sodium carbonate solution was added to wells of the 96-well plate. Absorbance of the samples was analyzed at 750 nm on a microplate reader (Thermo Electron, Spectronic Genesys 6, Madison, WI, USA). The results were expressed as gallic-acid-equivalent concentration (31.25–500 μg/mL). TFC was quantified as follows: 20 μL of 1N NaOH, 180 μL of 90% diethylene glycol, and 20 μL of each sample were mixed in a 96-well plate and incubated for 60 min in the dark. Then, absorbance was analyzed at 450 nm on the microplate reader. The results were expressed as naringin-equivalent concentration (15.625–200 μg/mL).

### Quantification of antioxidant activity, i.e., ABTS, and pancreatic lipase activity during the fermentation

The ABTS assay procedure was performed as described by Re et al. [19] with some modifications. A 7 mM ABTS solution was prepared by mixing with 2.45 mM potassium persulfate solution, and the reaction mixture was activated in a water bath for 20 min at 60°C. Then, the ABTS reaction was allowed to proceed for 12 h at room temperature in the dark to reach a stable state. The ABTS solution was diluted with deionized water to absorbance of 0.7 (± 0.02) at 734 nm. Each sample extract (5 mg/mL, 20 μL) was combined with 180 μL of the 7 mM ABTS solution and incubated for 6 min at room temperature in the dark. After that, the absorbance of the samples was analyzed at 734 nm on the microplate reader.

A pancreatic-lipase activity assay was conducted using the method of Ahn et al. [20] with minor modification. The enzyme solution was prepared by reconstitution of porcine pancreatic lipase in 0.1 M Tris-HCl buffer (pH 8). Five microliters of each sample was mixed with 90 μL of enzyme buffer. After incubation for 15 min at 37°C, 5 μL of 10 mM *p*-NPB was added to the enzyme reaction mixture. Pancreatic lipase activity was analyzed at 405 nm on the microplate reader after further incubation for 15 min at 37°C. The results were expressed as the relative pancreatic lipase activity (%) using the following formula: [(OD of negative control–OD of sample) ÷ OD of negative control] × 100; where negative control is dimethyl sulfoxide and OD is optical density at 405 nm.

### The animal experiment

Seven-week-old C57BL6J male mice were purchased from Daehan bio-link (Chungbuk, Republic of Korea). All mice were acclimated for 1 week under controlled conditions (temperature: 25 ± 2°C, relative humidity: 50 ± 5%, and 12 h light/dark cycle). The animals had free access to a normal diet (AIN-76A, Research Diets, Inc., NJ, USA) and water. After 1 week, the mice were randomly distributed into 5 groups: (1) group normal-diet (ND) (n = 11) was fed a normal diet for 6 weeks, (2) group HFD (named HD; n = 10) was fed 60 kcal% fat (D1242, Research Diets, Inc., NJ, USA) for 6 weeks, (3) group HFD with xenical administration (HX, 50 mg·kg^-1^·day^-1^) n = 10, 6 weeks, (4) group HFD with CLM administration (HCLM, 2 g·kg^-1^·day^-1^) n = 10, 6 weeks; and (5) group HFD with FCLM (CLM fermented for 60 h) administration (HFCLM, 2 g·kg^-1^·day^-1^) n = 8, 6 weeks. In this study, we used xenical as a positive control for antiobesity effects. Xenical, CLM, and FCLM dissolved with saline were orally administered everyday into the stomach with an oral zonde needle. And, the equal volume of saline were applied for ND and HD groups. During the experimental periods, the feed intake and the body weight of the mice were measured daily. All animal procedures were approved by the Institutional Animal Care and Use Committee (IACUC) of Daejeon University (Daejeon, Korea, decision # DJUARB2014-042).

For blood collection, the mice were sacrificed by cardiac puncture. Plasma (100 μL) was subjected to extraction with cold methanol (500 μL) on a MM400 mixer mill (Retsch®, Haan, Germany) with the frequency 30 s^-1^ for 5 min. The suspension was centrifuged at 12,578 *g* for 10 min at 4°C. The supernatant was filtered through a 0.2-μm PTEE filter and evaporated in a speed vacuum concentrator. The final concentration of the plasma was 5 mg/mL in methanol for the UPLC-Q-TOF-MS analysis.

### Measurement of biochemical parameters after administration of CLM, FCLM, or xenical to the mice with HFD-induced obesity

The alanine transaminase (ALT), creatinine, high-density lipoprotein cholesterol (HDL-c), low-density lipoprotein cholesterol (LDL-c), total cholesterol (TC), triacylglycerol (TG), and glucose were analyzed on an automatic blood chemical analyzer (Hitachi-720, Hitachi Medical, Tokyo, Japan). The leptin and insulin concentrations were determined with commercial kits (Abcam plc, Cambridge, UK).

### UPLC-Q-TOF-MS

The sample extracts were analyzed on a Waters Micromass Q-TOF Premier with a UPLC Acquity system (Waters Corp., Milford, MA, USA) equipped with a binary solvent delivery system, an autosampler, and a UV detector. We selected an Acquity UPLC BEH C18 column (100 mm × 2.1 mm × 1.7 μm particle size; Waters Corp.). The injection volume and flow rate were 5 μL and 0.3 mL/min, respectively. The column temperature was set to 37°C. Mobile phases consisted of 0.1% formic acid in water (solvent A) and 0.1% formic acid in acetonitrile (solvent B); the initial condition was 5% B for 1 min, then B was gradually increased to 100% during 9 min; the 100% B was maintained for 1 min and then decreased to 5% B during 3 min. The total run time was 14 min. The data from the Waters Q-TOF Premier Mass spectrometer (Micromass MS Technologies, Manchester, UK) were collected in the range 100–2,000 *m/z* in negative and positive ion modes. The source temperature was 100°C, and the flow of the desolvation gas (nitrogen) was set to 600 L/h at 300°C; the flow of the cone gas (nitrogen) was set to 50 L/h. The capillary voltage and cone voltage were set to 2.5 kV and 50 V, respectively. The data were collected with scan accumulation time of 0.2 s in the centroid mode. Leucine enkephalin (1 ppm) was used as the reference lock mass (*m/z* 554.2615 [−] and 556.2771 [+]) at the flow rate of 5 μL/min.

### Data processing and multivariate statistical analysis

The UPLC-Q-TOF-MS data were analyzed with MassLynx software (version 4.1, Waters Corp.), and raw data files were converted to the NetCDF format (*.cdf) using the MassLynx DataBridge (version 4.1, Waters Corp.). After that, peak detection, retention time correction, and alignment were performed in the MetAlign software package (http://www.metalign.nl). The resulting data were exported to an Excel file. Multivariate statistical analysis was performed using SIMCA-P+ software (version 12.0, Umetrics, Umea, Sweden). Differences among the experimental groups in ABTS, TPC, TFC, and pancreatic lipase activity were tested by analysis of variance (ANOVA) and Duncan’s multiple-range test in the PASW Statistics 18.0 software (SPSS Inc., Chicago, IL, USA). In the animal experiment, the clinical and histological results were evaluated by Student’s *t* test in PASW Statistics. A heatmap was constructed using the MeV software (http://www.tm4.org/) to visualize the differences in the metabolites. A box-whisker plot was built on the basis of means from the datasets in the Statistica 7 software (StatSoft Inc., Tulsa, OK, USA).

### Identification of metabolites by UPLC-Q-TOF-MS

To identify significantly different metabolites during fermentation, we used the variable importance in projection (VIP) value >0.7 and *p* values <0.05 in partial least squares-discriminant analysis (PLS-DA) model. A total of 40 metabolites were tentatively identified on the basis of their retention time, measured mass, elemental composition, and i-Fit in comparison with the standard compounds, in-house library, and references (S1 Table). Furthermore, we performed orthogonal PLS-DA (OPLS-DA) model to compare the compositions between two samples at the initial and end time of fermentation in CLM. To identify the significantly altered metabolites in plasma among the experimental groups in the three-dimensional PLS-DA model, we used VIP > 0.7 and *p* < 0.05. A total of 22 metabolites were identified, including 16 lysophosphatidylcholines (lysoPCs), 4 lysophosphatidylethanolamines (lysoPEs), and 2 unidentified metabolites (Table 1). Confirmation of the metabolites was performed in the Human Metabolome Database (HMDB, http://www.hmdb.ca/), high-resolution mass data (ppm), elemental composition analysis software, an in-house library, and references.

**Table 1:** The plasma metabolites significantly altered by CLM, FCLM, and xenical administration in the mouse model of HFD-induced obesity according to UPLC-Q-TOF-MS analysis.

No.	RT^a^	Tentative metabolites	Measured Mass (*m/z*)		MW^b^	HMDB formula^c^	Error (mDa)	PPM	Fold change^d^			
			Positive	negative					HD/ND	HCLM/HD	HFCLM/HD	HX/HD
1	7.26	-	288.2914	-	-	-	-	-	0.77	1.06	0.93	0.89
2	7.69	-	437.1937	-	-	-	-	-	0.81	0.94	1.31	0.89
3	8.01	LysoPC(14:0)	468.3107	452.2792	467	C22H46NO7P	2.8	6	0.93	0.8	0.87	1.22^#^
4	8.04	LysoPC(18:3)*	518.3263	502.2992	517	C22H46NO7P	2.3	4.4	0.52	0.85	0.93	1.37^#^
5	8.17	LysoPC(18:3)*	518.3259	502.2906	517	C22H46NO7P	0.7	1.4	0.66	0.99	1.11	1.33^#^
6	8.39	LysoPE(22:6)*	526.2955	524.2861	525	C27H44NO7P	-0.1	-0.2	1.43	0.88	1.07	0.88
7	8.41	LysoPC(22:6)*	568.3396	552.305	567	C30H50NO7P	-3.1	-5.5	1.51	0.81^#^	0.98	0.74^#^
8	8.45	LysoPC(18:2)*	520.3391	504.306	519	C26H50NO7P	3.5	6.7	0.83	1.03	1.14^#^	1.20^#^
9	8.47	LysoPC(20:4)*	544.3388	528.3116	543	C28H50NO7P	-3.8	-7	1.35	0.85	0.91	0.93
10	8.53	LysoPE(22:6)*	526.299	524.2785	525	C27H44NO7P	-1	-1.8	1.31	0.88	1.02	0.91
11	8.57	LysoPC(22:6)*	568.3409	552.3084	567	C30H50NO7P	1.1	1.9	1.42	0.84^#^	0.96	0.81^#^
12	8.57	LysoPE(18:0)	482.3263	466.3015	481	C23H48NO7P	1.5	3.1	0.57	0.89	0.95	1.22^#^
13	8.62	LysoPC(20:4)*	544.3397	528.3093	543	C28H50NO7P	0.5	0.9	1.37	0.86^#^	0.85^#^	0.92
14	8.65	LysoPC(18:2)*	520.34	504.3059	519	C26H50NO7P	1.6	3.1	0.77	1.01	1.08	1.23^#^
15	8.84	LysoPC(22:5)	570.3575	614.3306	569	C30H52NO7P	2.7	4.7	1.16	0.70^#^	0.86	0.71^#^
16	8.86	LysoPC(20:3)*	546.3574	530.3217	545	C28H52NO7P	4.2	7.7	0.94	0.58	0.61	0.76
17	9.03	LysoPC(20:3)*	546.356	530.3247	545	C28H52NO7P	-2.4	-4.4	0.92	0.53	0.51	0.81
18	9.38	LysoPC(18:1)	522.3546	506.3217	521	C26H52NO7P	-0.8	-1.5	0.95	0.92	0.95	1.06^#^
19	9.62	LysoPC(20:2)	548.3734	532.338	547	C28H54NO7P	-0.1	-0.2	0.8	0.71	0.81	1.59^#^
20	10.03	LysoPC(18:0)*	524.3711	508.3421	523	C26H54NO7P	2.6	4.6	1.33	1.01	1.14	0.90^#^
21	10.25	LysoPC(18:0)*	524.3712	508.3445	523	C26H54NO7P	0.2	0.4	1.18	1.03	1.1	0.93

Metabolites selected by variable important in the projection (VIP) > 0.7 and *p* value < 0.05 from PLS-DA model.

^a^Retention time.

^b^Molecular weight.

^c^HMDB: The Human Metabolome Data Base (http://hmdb.ca/).

^d^Relative levels of metabolites were converted into fold changes.

*This means the 2 forms of lysoPC with the fatty acyl groups at *sn*-1 or *sn*-2 on the glycerol backbone.

^#^a significant difference between the HD group and a treatment group (HCLM, HFCLM, or HX, *p* value < 0.05).

LysoPC: lysophosphatidylcholine; LysoPE: lysophosphatidylethanolamine.

ND (normal-diet group), HD (high-fat diet group), HCLM (HFD with CLM administrated group), HFCLM (HFD with FCLM administrated group), HX (HFD with xenical administrated group).

## Results

### Metabolite variations in CLM during fermentation

**Fig 2:**
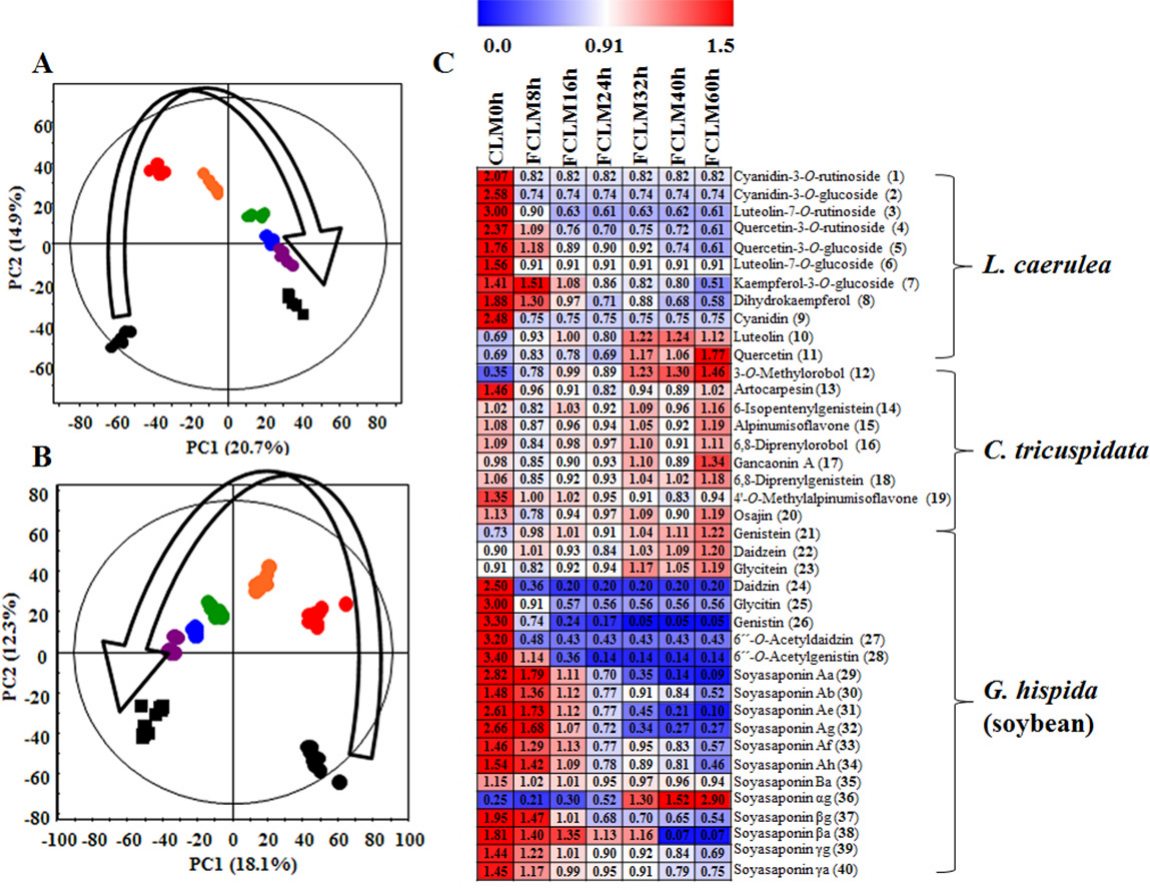
Principal component analysis (PCA; A: positive mode, B: negative mode) score plots and heatmap (C) derived from UPLC-Q-TOF-MS data on CLM during fermentation; ●CLM0h (unfermented CLM), ●FCLM8h (CLM fermented for 8 h), ●FCLM16h (CLM fermented for 16 h), ●FCLM24h (CLM fermented for 24 h), ●FCLM32h (CLM fermented for 32 h), ●FCLM40h (CLM fermented for 40 h), ■FCLM60h (CLM fermented for 60 h). Each data point shown on the heatmap was normalized by the mean values of each set.

These changes were analyzed by UPLC-Q-TOF-MS in positive and negative modes with multivariate analysis. According to principal component analysis (PCA) score plots (Fig 2A and 2B), CLM was clearly separated and grouped on the basis of the fermentation time intervals (0, 8, 16, 24, 32, 40, and 60 h). The PCA score plot of positive mode (Fig 2A) showed R^2^X_(cum)_ = 0.56 and Q^2^_(cum)_ = 0.444, and the negative mode (Fig 2B) showed R^2^X_(cum)_ = 0.523 and Q^2^_(cum)_ = 0.33. In this study, anthocyanin, anthoxanthin glycosides [cyanidin-3-*O*-rutinoside (**1**), cyanidin-3-*O*-glucoside (**2**), quercetin-3-*O*-rutinoside (**4**), quercetin-3-*O*-glucoside (**5**), luteolin-7-*O*-rutinoside (**3**), and luteolin-7-*O*-glucoside (**6**)] and their aglycones [cyanidin (**9**), luteolin (**10**), and quercetin (**11**)] were identified and were found to have originated from *L*. *caerulea*. Several *C*. *tricuspidata-*specific flavonoids including anthoxanthin, prenylflavonoid, and isoflavone were also identified. In addition, 3 forms of isoflavones such as acetylglucosides [6''-*O*-acetyldaidzin (**27**) and 6''-*O*-acetylgenistin (**28**)], *β*-glucosides [daidzin (**24**), glycitin (**25**) and genistin (**26**)], their aglycones [genistein (**21**), daidzein (**22**), and glycitein (**23**)], and soyasaponins were detected and were found to have originated from the soybean. The heatmap shows the patterns of changes in metabolites of CLM by the fermentation time intervals (Fig 2C). In particular, most of flavonoid glycosides were decreased at the early stage of the fermentation, while their aglycones including luteolin (**10**), quercetin (**11**), genistein (**21**), daidzein (**22**), and glycitein (**23**) were increased during the fermentation. Most of the metabolites derived from *C*. *tricuspidata* were slightly increased or not changed.

**Fig 3:**
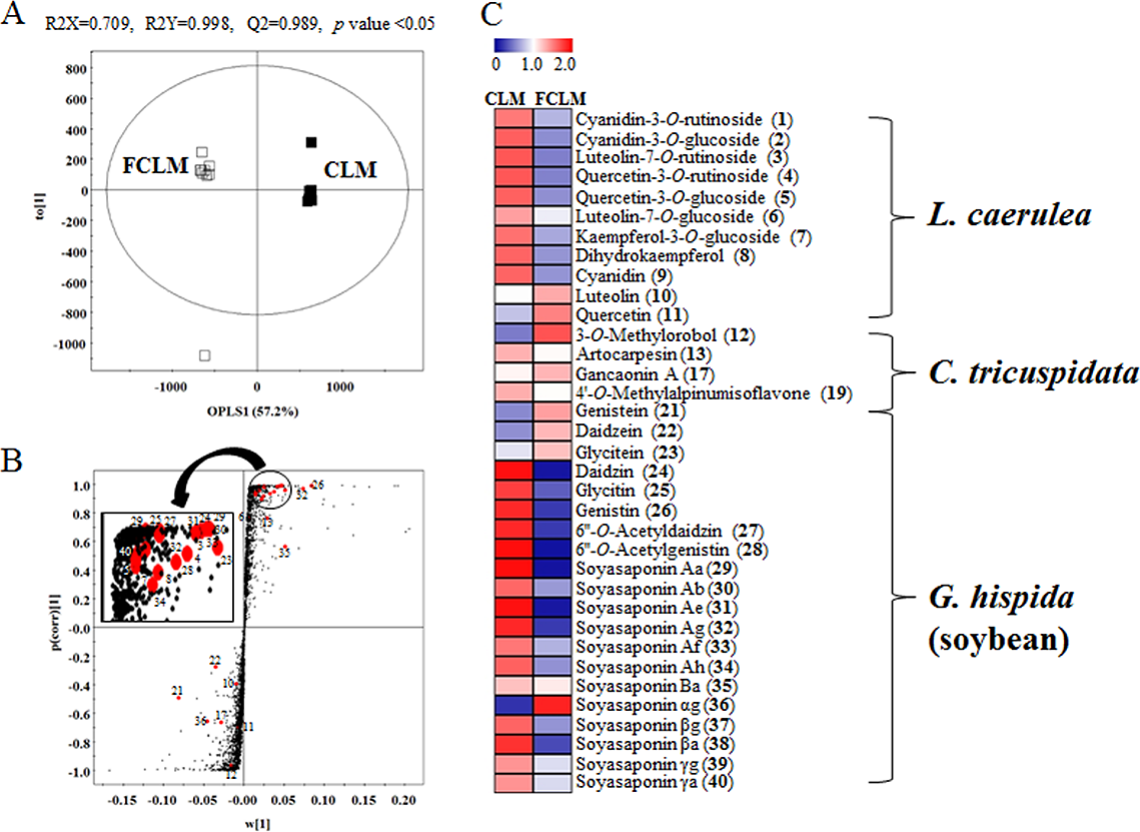
**OPLS-DA score plot (A), S-plot (B), and heatmap (C) derived from comparison of UPLC-Q-TOF-MS data sets between CLM and FCLM**. Each data point shown on the heatmap was normalized by the mean values of each set.

To compare the composition of CLM and FCLM, we performed OPLS-DA analysis (Fig 3A). CLM and FCLM were clearly separated with R^2^X_(cum)_ = 0.709, R^2^Y_(cum)_ = 0.998, Q^2^_(cum)_ = 0.989, and *p* value < 0.05. The S-plot from the OPLS-DA model (Fig 3B) showed a distribution of selected metabolites between CLM and FCLM. According to the heatmap analysis (Fig 3C), several flavonoid glycoside forms [cyanidin-3-*O*-rutinoside (**1**), cyanidin-3-*O*-glucoside (**2**), luteolin-7-*O*-rutinoside (**3**), quercetin-3-*O*-rutinoside (**4**), quercetin-3-*O*-glucoside (**5**), luteolin-7-*O*-glucoside (**6**), kaempferol-3-*O*-glucoside (**7**), daidzin (**24**), glycitin (**25**), genistin (**26**), 6''-*O*-acetyldaidzin (**27**), and 6''-*O*-acetylgenistin (**28**)] and soyasaponins were present at a higher concentration in CLM than in FCLM. In contrast, the levels of their aglycones [luteolin (**10**), quercetin (**11**), genistein (**21**), daidzein (**22**), and glycitein (**23**)], which formed due to the fermentation by *B*. *subtilis*, were elevated in FCLM.

### ABTS, pancreatic lipase activity, TPC, and TFC in CLM during the fermentation

**Fig 4:**
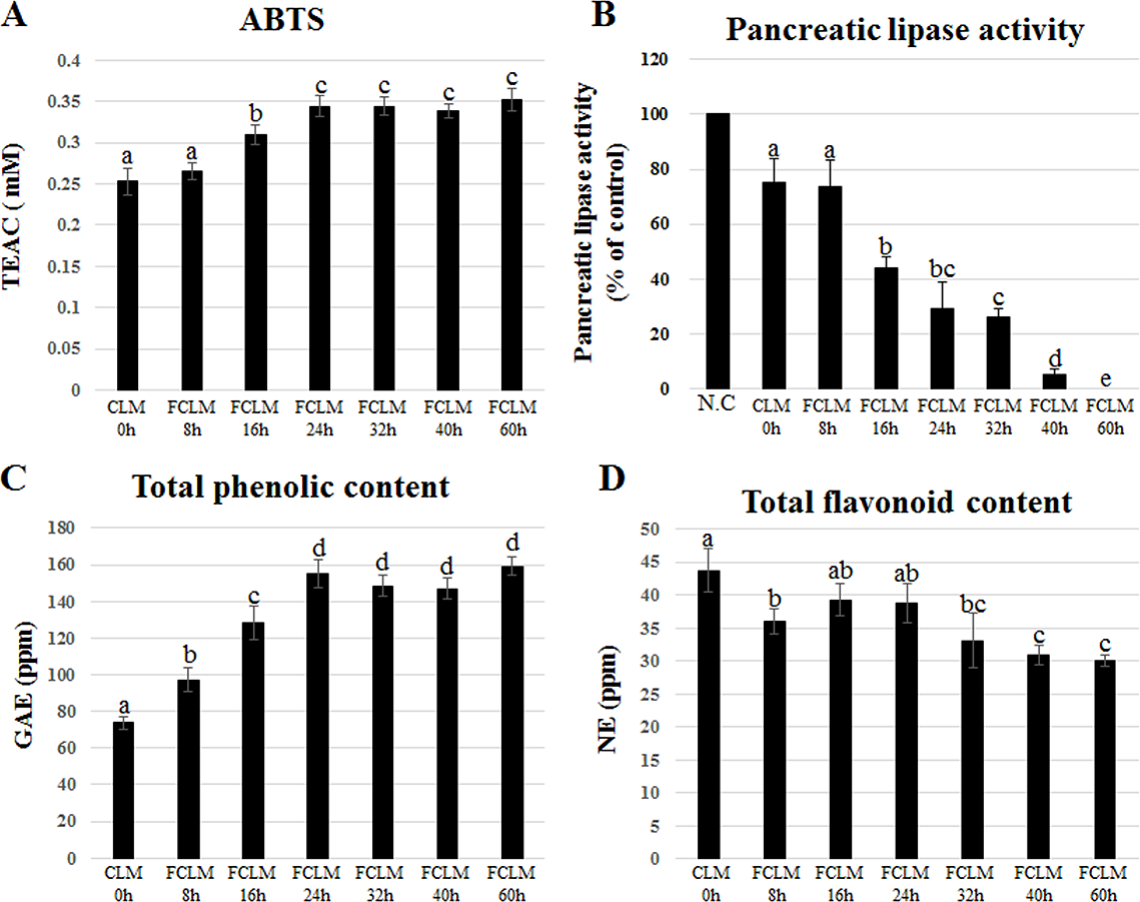
**The results of analysis of antioxidant (A) and pancreatic lipase activities (B), TPC (C), and TFC (D) of CLM during fermentation**; CLM0h (unfermented CLM), FCLM8h (CLM fermented for 8 h), FCLM16h (CLM fermented for 16 h), FCLM24h (CLM fermented for 24 h), FCLM32h (CLM fermented for 32 h), FCLM40h (CLM fermented for 40 h), FCLM60h (CLM fermented for 60 h). Different letters are significantly different according to Duncan’s multiple-range test (*p* < 0.05).

Various tests for ABTS, pancreatic lipase activity, TPC, and TFC were conducted at different time points during the fermentation (0, 8, 16, 24, 32, 40, and 60 h). The antioxidant activity, i.e., ABTS, was gradually increased from 0 to 24 hours and then maintained approximately the same until 60 hours (Fig 4A). As shown in Fig 4B, pancreatic lipase activity was gradually inhibited during the fermentation. The TPC results were similar to the ABTS results. TPC gradually increased from hour 0 to hour 24 and then stayed approximately the same until hour 60 (Fig 4C). In contrast, TFC slightly decreased during the fermentation (Fig 4D). According to the results of ABTS and pancreatic lipase activity, FCLM had possessed significantly higher activities than CLM.

### Clinical data and histopathological effects of CLM, FCLM, and xenical administration to mice with HFD-induced obesity

**Fig 5:**
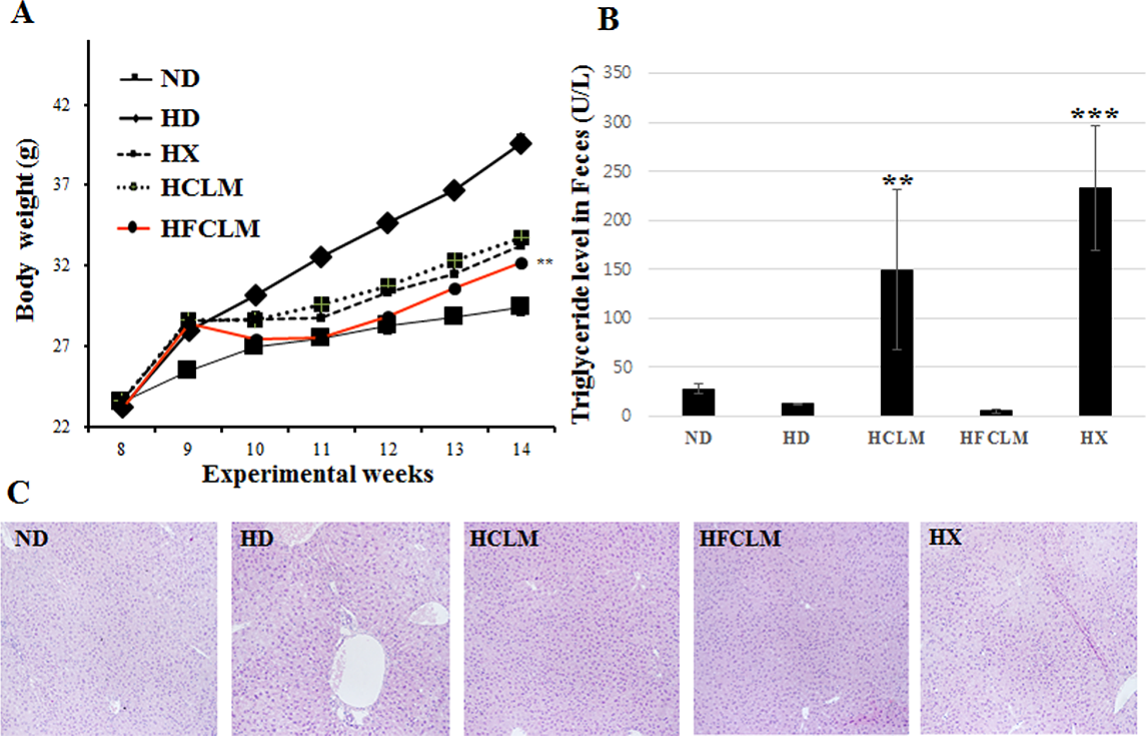
**The body weight change (A), triglyceride (TG) level in feces (B), and alterations in the histopathology of the liver tissues stained with hematoxylin and eosin (C) of high-fat diet (HFD) groups (mouse model of HFD-induced obesity) receiving CLM, FCLM, or xenical**; ND (normal-diet group), HD (high-fat diet group), HX (HFD with xenical administrated group), HCLM (HFD with CLM administrated group), HFCLM (HFD with FCLM administrated group). The statistical analysis was performed by an independent *t* test in comparison with the HD group (**p value < 0.01, ***p value < 0.001).

After HFD feeding for 6 weeks, the body weight significantly increased in the HD group compared to the ND group. The oral administration of CLM, FCLM, and xenical suppressed the weight gain (Fig 5A). In particular, the HFCLM group showed a smaller body weight gain than did the other groups (HX and HCLM). Besides, the weights of organs and tissues were increased by HFD. These changes were also inhibited by CLM, FCLM, and xenical administration. FCLM administration showed the strongest attenuation of the increase of the adipocyte area by HFD (Table 2). Among the biochemical parameters of plasma, the levels of TC, TG, HDL-c, glucose, ALT, insulin, and leptin were increased by HFD, whereas CLM, FCLM, and xenical administration inhibited these changes. In particular, the upregulation of TC, TG, ALT, insulin, and leptin was significantly attenuated by CLM administration. Because the mechanism of fat excretion under the influence of xenical administration is well known, the levels of TG in feces were also measured in the experimental groups. As shown in Fig 5B, the HX group showed a higher level of TG than did the other groups. The HCLM group showed a result similar to that in the HX group, while the HFCLM group did not show significant TG excretion in feces. According to the results of H&E staining of the liver, lipid vacuoles did not appear in the liver in groups HCLM, HFCLM, and HX in contrast to the HD group.

**Table 2:** The effect of CLM, FCLM, and xenical administration on histological and blood biochemical parameters in the mouse model of HFD-induced obesity.

	Experimental groups				
Parameters	ND	HD	HCLM	HFCLM	HX
**Weight of tissues**					
Subcutaneous fat (g)	0.33±0.02	1.59±0.04^#^	1.00±0.09***	1.03±0.13***	0.90±0.09***
**Adipose tissue**					
Adipocyte area (μm)	49.40±1.65	128.20±1.83^#^	77.10±0.77***	70.30±1.08***	76.50±1.22***
Epididymal adipose tissue (g)	0.66±0.05	2.72±0.06^#^	1.81±0.11***	1.85±0.23**	1.68±0.10***
Adipose tissue (g)	0.23±0.03	1.11±0.03^#^	0.83±0.06***	0.84±0.10*	0.75±0.05***
**Biochemical parameters**					
Total cholesterol (mg/dL)	157.30±13.34	214.30±9.89^#^	192.00±5.69*	200.60±10.31	201.40±5.63
Triacylglycerol (mg/dL)	97.60±7.05	193.90±19.50^#^	89.90±6.63***	91.60±5.85***	107.80±3.07***
LDL-cholesterol (mg/dL)	30.60±2.89	36.40±5.97	31.10±2.09	31.80±1.87	28.90±3.25
HDL-cholesterol (mg/dL)	84.90±3.23	104.90±3.16^#^	104.30±3.63	109.00±4.09	86.00±1.71***
Glucose (mg/dL)	131.10±13.60	204.70±12.25^#^	196.10±13.01	202.90±10.70	188.70±13.28
ALT (U/L)	29.80±1.85	41.60±1.54^#^	25.60±1.35***	29.50±1.79***	33.70±2.06**
Insulin (ng/ml)	2935.60±339.10	3466.20±427.50^#^	2370.60±227.00*	3001.20±217.40	2472.55±193.50*
Leptin (ng/ml)	0.23±0.05	1.10±0.27^#^	0.37±0.08*	0.85±0.15	0.47±0.22*

The statistical analysis was performed by an independent *t* test in comparison with the HD group (^#^*p* value < 0.05, *p value < 0.05, **p value < 0.01, ***p value < 0.001).

ND (normal-diet group), HD (high-fat diet group), HCLM (HFD with CLM administrated group), HFCLM (HFD with FCLM administrated group), HX (HFD with xenical administrated group).

### Metabolite profiling of plasma by UPLC-Q-TOF-MS

We performed this analysis to identify possible differences in endogenous metabolites among the experimental groups, with multivariate statistical analysis. According to the three-dimensional PLS-DA score plot, each experiment group was clearly separated from the other groups (*p* value < 0.05; S1 Fig). The relative levels of the metabolites different among the experimental groups were converted into fold changes (Table 1). The levels of most lysoPCs and lysoPEs were affected by HFD and showed a tendency for restoration during CLM, FCLM, and xenical administration. In the comparison of the administration of CLM and FCLM, the levels of lysoPCs with C18:2 and C18:3 were decreased by FCLM administration, while lysoPCs with C22:5 and C22:6, and lysoPE(22:6) were decreased by CLM administration in the plasma of HFD-fed mice. LysoPC(20:4) and lysoPE(20:0) showed a tendency for recovery during administration of either CLM or FCLM.

**Fig 6:**
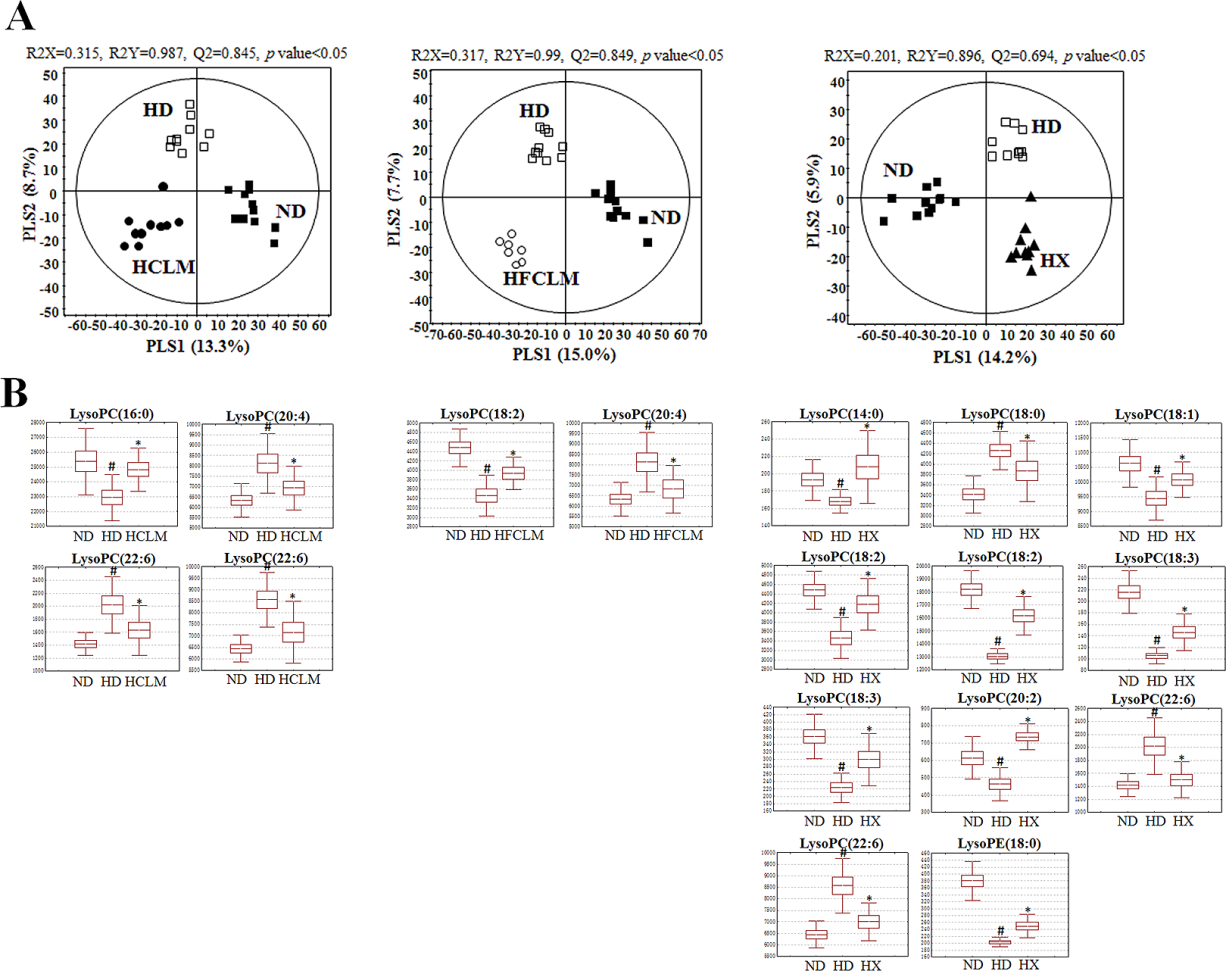
**PLS-DA score plots (A) and box-whisker plots (B) of statistically significant metabolites showing a tendency for restoration as a result of administration of CLM, FCLM, or xenical in the mouse model of high-fat diet (HFD)-induced obesity**; ND (normal-diet group), HD (high-fat diet group), HX (HFD with xenical administrated group), HCLM (HFD with CLM administrated group), HFCLM (HFD with FCLM administrated group). The statistical analysis was performed by an independent *t* test in comparison with the HD group (^#^*p* value < 0.05, *p value < 0.05).

To confirm the effect of CLM, FCLM, and xenical on plasma metabolites changed by HFD, PLS-DA models were applied, respectively (Fig 6A). The levels of metabolites that were significantly restored by CLM, FCLM, or xenical administration are shown as box-whisker plots in Fig 6B. The levels of LysoPCs with C16:0 and C22:6 were significantly altered by CLM administration, while the level of lysoPC(18:2) was significantly restored by FCLM administration. The lysoPC(20:4) was also significantly restored by administration of either CLM or FCLM. By contrast, the levels of 11 metabolites including lysoPCs with C14:0, C18:0, C18:1, C20:2, and the 2 forms of C18:2, C18:3, and C22:6 as well as lysoPE(18:0) were significantly restored in the HX group.

## Discussion

In this study, CLM and FCLM showed different biological activities according to the differences in components during fermentation *in vitro* and the significant differences in some plasma metabolites between groups HCLM and HFCLM in our metabolite profiling. Prior to this experiment, we selected a mixture of *C*. *tricuspidata*, *L*. *caerulea*, and soybean for fermentation because these natural products are already known for effects related to antiobesity properties and each contains distinct ingredients such as anthocyanins, anthoxanthins, prenylated flavonoids, isoflavones, and soyasaponins [6–11]. With these various flavonoids, we expected not only that the mixture of these natural products would show enhanced antiobesity effects in comparison with the stand-alone natural products but also that the fermentation process would have synergistic effects resulting from numerous fermentation products originating from these different natural products. Because *C*. *tricuspidata* and *L*. *caerulea* were already known to have antimicrobial activity [21,22], it was necessary to consider the mixing ratio of these natural products. We determined the optimal fermentation conditions [*C*. *tricuspidata* (1.5 g, 10% w/w of soybean), *L*. *caerulea* (0.3 g, 1% w/w of soybean), and soybean (15 g) fermented for 60 h] in consideration of both the enhanced antiobesity effects and growth of *B*. *subtilis* in a preliminary test with various sample concentrations and periods of fermentation (data not shown).

Our *in vitro* data showed that the concentrations of various natural flavonoid glycosides derived from *C*. *tricuspidata*, *L*. *caerulea*, and soybean were decreased by fermentation, while their aglycones including luteolin, quercetin, daidzein, glycitein, and genistein were increased during the fermentation (Fig 2C). These increased levels of aglycones are well known to be associated with fermentation [12,13]. Most flavonoids occur as *O*-glycosides in natural products [23]. Consequently, various flavonoid glycosides were hydrolyzed into their aglycones by the catalytic action of *β*-glucosidase from *B*. *subtilis* during the fermentation [24]. This phenomenon reflected the result of TFC. The decrease of TFC remained to the end of fermentation, which is what typically occurs in the fermentation process [25–27]. Increased consumption of various aglycones is beneficial for human health; their activities (including antioxidant, anti-inflammatory, and antiobesity properties) depend on both the position and number of hydroxyl groups [23]. Both antioxidant and pancreatic-lipase-inhibitory activities of CLM were increased during the fermentation due to the increase in the levels of aglycones. The level of the pancreatic lipase activity *in vitro* highly correlates with antiobesity effects *in vivo* (Fig 4). Pancreatic lipase inhibition suppresses absorption of dietary fats including TG and phospholipids in the intestinal tract. Decomposed fats tend to be excreted in feces, and this phenomenon correlates with weight loss [20]. FCLM showed improved inhibition of pancreatic lipase due to the upregulation of fermentation products including flavonoid aglycones. Judging by these results, we hypothesized that FCLM (with its large quantities of flavonoid aglycones) has more potent antiobesity effects than does CLM in the mouse model of HFD-induced obesity.

Accordingly, the antiobesity effects by CLM and FCLM administration were evaluated in those mice. The weight of body, organs, and adipose tissue and some biochemical parameters were increased by HFD. On the other hand, administration of either CLM or FCLM had antiobesity effects with some differences in the attenuation of the HFD-induced alterations. The levels of TG in feces, TC, creatinine, and leptin were significantly decreased by CLM administration, while FCLM administration was more effective at reducing the body weight and the adipocyte area than were the other treatment groups (HX and HCLM). However, the weights of organs and tissues, and the levels of TG and ALT were similarly improved by administration of CLM and FCLM. The above-mentioned differences were probably caused by changes in chemical composition of CLM after fermentation [26]. In spite of these metabolite variations, some similar outcomes did occur to result from the metabolism of these ingredients *in vivo*. According to other reports, flavonoid aglycones are absorbed faster and metabolized more effectively than flavonoid glucoside forms are. As for dietary flavonoid glucoside forms, they act in a way similar to the mechanism of action of their flavonoid aglycones as a result of metabolism by gut microflora when they are consumed for a long period [1,27]. According to these observations, flavonoid glycosides from CLM were likely degraded into their aglycones by some gut microflora in the mice; therefore, CLM administration did occur to show a tendency for restoration of various biochemical parameters similar to that after FCLM administration. In the HX group (positive control group), the clinical and histological parameters including the TG level in feces, body weight, the weight of organs, and adipocyte area were significantly normalized in comparison with the HD group.

On the basis of these results, we also found (by metabolite profiling) that CLM and FCLM administration attenuated HFD-induced alterations of plasma metabolites. Many researchers reported that the levels of lysoPCs are remarkably changed in the plasma of HFD-fed mice [14–17,28]. LysoPCs are important signaling molecules with biological functions including cellular proliferation, penetration of tumor cells, and inflammation [15]. Furthermore, Pietiläinen and colleagues found that obesity is strongly associated with a change in lysoPCs levels, and these changes are associated with deleterious alterations in the lipid metabolism [29]. In the present study, the levels of lysoPCs and lysoPEs that were significantly changed by HFD were altered by the intake of CLM, FCLM, and xenical. Xenical administration showed normalization of a relatively greater number of plasma metabolites than either HCLM or HFCLM groups. Lysophospholipid activity is dependent on lipid structure. In other words, the differences in the restoration of the levels of these lysophospholipids between CLM and FCLM administration did occur to depend on the length of the carbon chain and the number of double bonds in the fatty acyl group of lysoPCs and lysoPEs. The levels of lysophospholipids with the fatty acid containing 20 carbons [lysoPE(20:0) and lysoPC(20:4)] and lysoPC(22:5) showed recovery patterns with both CLM and FCLM administration. In particular, lysoPCs with C18:0 and C18:1, and lysoPE(18:0) were not significantly altered after administration of either CLM or FCLM, while lysoPC(18:2) and lysoPC(18:3), which contain more double bonds, showed a tendency for normalization as a result of FCLM administration. In contrast, decohexaenoyl-lysophospholipids [lysoPC(22:6) and lysoPE(22:6)] were improved only by CLM administration in the plasma of the HFD-fed mice. Moreover, the levels of lysoPCs with C16:0 and C22:6 were significantly restored by CLM administration, while FCLM administration significantly improved the level of lysoPC(18:2). These plasma metabolites may explain the similar but different antiobesity effects of CLM and FCLM administration. However, our results are insufficient to fully explain the relations between metabolites and antiobesity effects because our study is limited to the changes in the levels of lysoPCs and lysoPEs in plasma. Further studies on the difference between CLM and FCLM administration in antiobesity effects are needed to confirm metabolic pathway in depth *via* comprehensive MS analysis for various metabolites (e.g. amino acids, sugars, organic acids, fatty acids, and other lipids) in other biofluids and organ tissues.

In conclusion, we tested a new functional fermented food product for antiobesity effects and tried to explain the enhancement of antiobesity effects due to fermentation of CLM not only *in vitro* (biochemical parameters) but also *in vivo* (in a mouse model of obesity based on metabolite profiling). We demonstrated that antioxidant and pancreatic-lipase-inhibitory activities of CLM were increased during fermentation due to flavonoid aglycones including genistein, daidzein, glycitein, luteolin, and quercetin. Moreover, several lysophospholipids in the plasma of the HFD-fed mice showed differences in the tendency to normalize after CLM and FCLM administration, based on to the length of the carbon chain and the number of double bonds. The proposed MS-based metabolomics method may provide some clues regarding the antiobesity effects of some drugs or foods. In this study, we suggest that metabolomics provides not only to explain metabolite changes, but also provides a better understanding of the differences in metabolic activity.

## Supporting Information

S1 Dataset Raw data excel file of Figures and Tables. (XLSX)

S1 Fig **3D PLS-DA score plot (A) and box-whisker plots (B) of altered plasma metabolites in HFD-fed obese mice analyzed by UPLC-Q-TOF-MS**. The statistical analysis was performed by an independent *t*-test (*p value < 0.05, **p value < 0.01, ***p value < 0.001).
(TIF)

S1 Table Tentative identification of significantly altered metabolites of CLM during fermentation analyzed by UPLC-Q-TOF-MS combined with multivariate analysis. (PDF)
