# Supplementary figures and images for: Comparison of Metabolites Variation and Antiobesity Effects of Fermented versus Nonfermented Mixtures of Cudrania tricuspidata, Lonicera caerulea, and Soybean According to Fermentation In Vitro and In Vivo
Source: PLoS One. 2016 Feb 5;11(2):e0149022. doi: 10.1371/journal.pone.0149022 (PMC4743955; doi:10.1371/journal.pone.0149022)

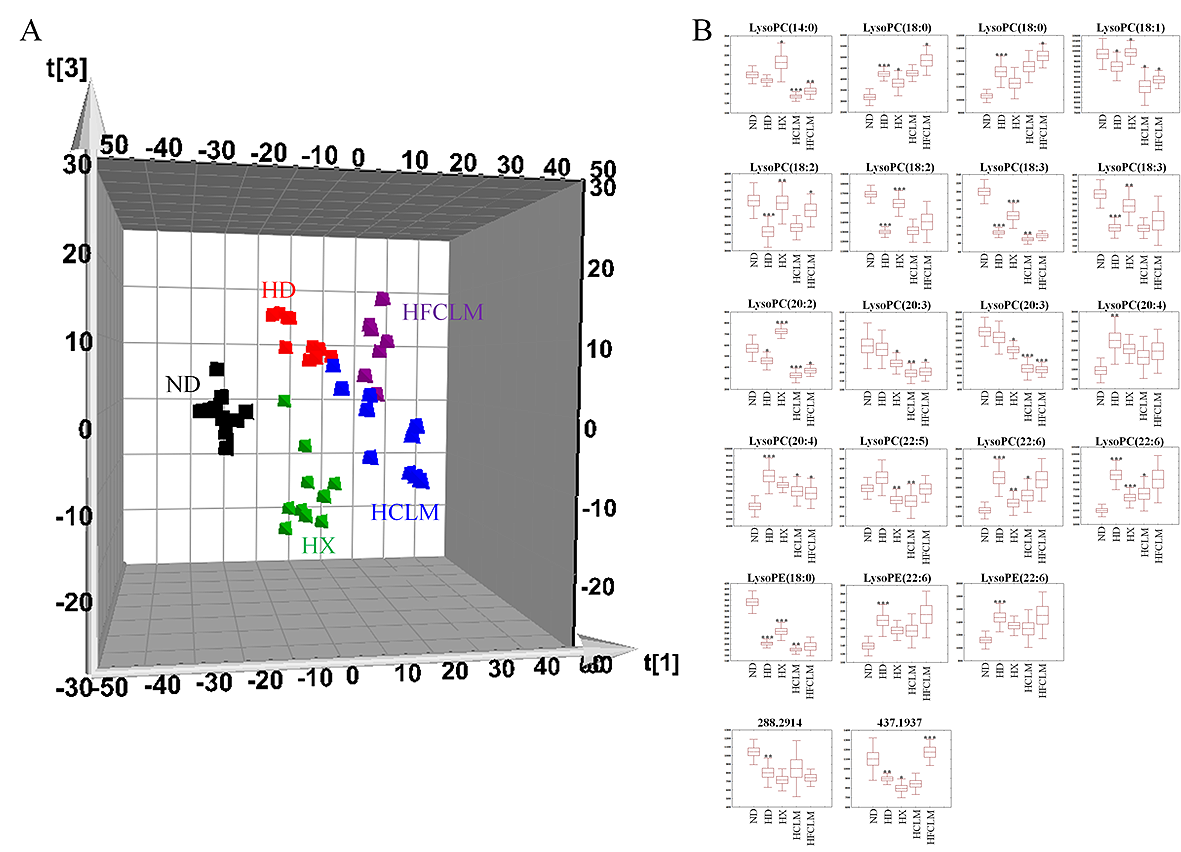

Supplement: S1 Fig — 3D PLS-DA score plot (A) and box-whisker plots (B) of altered plasma metabolites in HFD-fed obese mice analyzed by UPLC-Q-TOF-MS. The statistical analysis was performed by an independent t-test (*p value < 0.05, **p value < 0.01, ***p value < 0.001). (TIF) [file pone.0149022.s002.tif]
