# Supplementary material for: Comparison of Metabolites Variation and Antiobesity Effects of Fermented versus Nonfermented Mixtures of Cudrania tricuspidata, Lonicera caerulea, and Soybean According to Fermentation In Vitro and In Vivo
Source: PLoS One. 2016 Feb 5;11(2):e0149022. doi: 10.1371/journal.pone.0149022 (PMC4743955; doi:10.1371/journal.pone.0149022)
Supplement: S1 Table — (PDF) [file pone.0149022.s003.pdf]

S1 Table. Tentative identification of significantly altered metabolites of CLM (*C. tricuspidata* and *L. caerulea* with soybean mixture) during fermentation analyzed by UPLC-Q-TOF-MS combined with multivariate analysis

No.	RT[a]	Metabolites	Biological Source	Identified ion (m/z)	Adduct	Elemental composition (Formula)	VIP1	VIP2	Error (ppm)	i-Fit (Norm)	I.D[b]
1	3.27	Cyanidin-3-*O*-rutinoside	*L. caerulea*	595.1683	$[M^+]$	C27H31O15	1.56	1.65	-7.7	3.8	7
2	3.30	Cyanidin-3-*O*-glucoside	*L. caerulea*	449.1084	$[M^+]$	C21H21O11	1.55	1.63	-0.9	4.2	7
3	3.42	Luteolin-7-*O*-rutinoside	*L. caerulea*	593.1529	$[M-H]^-$	C27H29O15	1.39	1.79	6.6	5.9	5.25
4	3.88	Quercetin-3-*O*-rutinoside	*L. caerulea*	609.1467	$[M-H]^-$	C27H29O16	1.64	1.67	-1.3	6.7	7
5	4.03	Quercetin-3-*O*-glucoside	*L. caerulea*	463.0904	$[M-H]^-$	C21H19O12	1.70	1.45	3.0	5.3	7
6	4.06	Luteolin-7-*O*-glucoside	*L. caerulea*	447.1011	$[M-H]^-$	C21H19O11	1.09	1.49	-0.4	3.7	5.25
7	4.25	Kaempferol-3-*O*-glucoside	*L. caerulea*	447.0953	$[M-H]^-$	C21H19O11	2.22	1.57	-3.1	2.1	5.25
8	4.67	Dihydrokaempferol	*L. caerulea*	287.0564	$[M-H]^-$	C15H11O6	1.96	1.59	7.0	3.4	7
9	3.80	Cyanidin	*L. caerulea*	287.0566	$[M^+]$	C15H11O6	1.56	1.65	-9.1	5.1	7
10	5.02	Luteolin	*L. caerulea*	285.0409	$[M-H]^-$	C15H9O6	0.98	0.93	-0.4	0.1	7
11	5.12	Quercetin	*L. caerulea*	301.0344	$[M-H]^-$	C15H10O7	1.26	0.99	9.3	1.7	7
12	5.65	3-*O*-Methylorobol	*C. tricuspidata*	299.0562	$[M-H]^-$	C16H11O6	2.06	1.50	6.7	2.9	5.25
13	6.94	Artocarpesin	*C. tricuspidata*	353.0999	$[M-H]^-$	C20H17O6	1.03	1.64	-7.6	0.3	5.25
14	7.43	6-Isopentenylgenistein	*C. tricuspidata*	337.1073	$[M-H]^-$	C20H17O5	0.87	0.79	-3.9	0.7	5.25
15	8.02	Alpinumisoflavone	*C. tricuspidata*	335.0925	$[M-H]^-$	C20H15O5	1.13	1.55	2.4	0.0	5.25
16	8.41	6,8-Diprenylorobol	*C. tricuspidata*	421.1649	$[M-H]^-$	C25H25O6	1.28	1.24	1.2	0.3	5.25
17	8.56	Gancaonin A	*C. tricuspidata*	351.1218	$[M-H]^-$	C21H19O5	1.13	0.93	0.5	0.2	5.25
18	8.93	6,8-Diprenylgenistein	*C. tricuspidata*	405.1689	$[M-H]^-$	C25H25O5	1.01	1.17	-3.9	0.8	5.25
19	9.37	4'-*O*-Methylalpinumisoflavone	*C. tricuspidata*	351.1202	$[M+H]^+$	C21H19O5	1.69	1.41	-4.6	1.3	5.25
20	9.54	Osajin	*C. tricuspidata*	403.1545	$[M-H]^-$	C25H23O5	0.75	0.87	1.7	0.5	5.25
21	5.55	Genistein	*C. tricuspidata* *G. hispida*	269.0452	$[M-H]^-$	C15H9O5	0.95	0.87	-3.7	0.0	7
22	4.93	Daidzein	*G. hispida*	253.0499	$[M-H]^-$	C15H9O4	1.55	1.41	2.0	0.1	7
23	5.04	Glycitein	*G. hispida*	285.0732	$[M+H]^+$	C16H13O5	1.55	1.30	-2.5	1.4	7
24	3.71	Daidzin	*G. hispida*	415.1033	$[M-H]^-$	C21H19O9	1.33	1.76	-9.9	0.8	7
25	3.76	Glycitin	*G. hispida*	445.1154	$[M-H]^-$	C22H21O10	1.47	1.77	4.0	6.4	7
26	4.15	Genistin	*G. hispida*	431.0977	$[M-H]^-$	C21H19O10	1.33	1.81	0.7	1.0	7
27	4.43	6"-*O*-Acetyldaidzin	*G. hispida*	457.1137	$[M-H]^-$	C23H21O10	1.31	1.75	3.9	2.4	7
28	4.88	6"-*O*-Acetylgenistin	*G. hispida*	473.1088	$[M-H]^-$	C23H21O11	1.58	1.77	-2.1	0.6	7
29	5.60	Soyasaponin Aa	*G. hispida*	1363.6693	$[M-H]^-$	C64H99O31	2.19	1.68	-	-	5.25
30	5.70	Soyasaponin Ab	*G. hispida*	1435.7114	$[M-H]^-$	C67H103O33	2.08	1.48	-	-	5.25
31	5.81	Soyasaponin Ae	*G. hispida*	1201.5878	$[M-H]^-$	C58H89O26	2.20	1.66	6.6	3.9	5.25
32	5.86	Soyasaponin Ag	*G. hispida*	1171.5758	$[M-H]^-$	C57H87O25	2.14	1.68	-	-	5.25
33	5.90	Soyasaponin Af	*G. hispida*	1273.6193	$[M-H]^-$	C61H93O28	2.01	1.42	-	-	5.25
34	5.97	Soyasaponin Ah	*G. hispida*	1243.6068	$[M-H]^-$	C60H91O27	2.08	1.48	-	-	5.25
35	6.23	Soyasaponin Ba	*G. hispida*	957.5134	$[M-H]^-$	C48H77O19	0.92	0.70	5.0	4.4	5.25
36	6.66	Soyasaponin αg	*G. hispida*	1083.5846	$[M-H]^-$	C54H83O22	1.45	1.15	-	-	5.25
37	6.75	Soyasaponin βg	*G. hispida*	1067.5476	$[M-H]^-$	C54H83O21	2.02	1.59	1.8	5.5	5.25
38	6.89	Soyasaponin βa	*G. hispida*	1037.5392	$[M-H]^-$	C53H81O20	1.47	1.10	2.5	3.2	5.25
39	7.02	Soyasaponin γg	*G. hispida*	921.4888	$[M-H]^-$	C48H73O17	1.97	1.47	5.2	7.3	5.25
40	7.10	Soyasaponin γa	*G. hispida*	891.4804	$[M-H]^-$	C47H71O16	1.66	1.35	9.3	4.9	5.25

[a]RT: Retention time

[b] Metabolite identification scores were followed by Sumner et al, Metabolomics, 2014, 10(6), 1047-1049. Significantly altered metabolites were selected according to VIP > 0.7 and p value < 0.05 from PLS-DA model.

References

1. Sumner LW, Lei Z, Nikolau BJ, Saito K, Roessner U, Trengove R (2014) Proposed quantitative and alphanumeric metabolite identification metrics. Metabolomics. 10: 1047-1049. DOI: 10.1007/s11306-014-0739-6
